# Relationship between Plasma and Saliva Urea Nitrogen Concentrations in New Zealand Red Deer Calves (*Cervus elaphus*)

**DOI:** 10.3390/ani14172565

**Published:** 2024-09-03

**Authors:** E. Wilson, A. Fleming, M. Vollebregt, P. Gregorini

**Affiliations:** Department of Agricultural Sciences, Lincoln University, Christchurch 7674, New Zealand; anita.fleming@lincoln.ac.nz (A.F.); vollebregtmatthew@gmail.com (M.V.); pablo.gregorini@lincoln.ac.nz (P.G.)

**Keywords:** saliva urea nitrogen, plasma urea nitrogen, deer, diet, date

## Abstract

**Simple Summary:**

The relationship between the saliva urea N (SUN) and plasma urea N (PUN) concentrations was investigated in red deer calves (*n* = 23) over a five-month period with the objective of creating an easy tool to quickly evaluate circulating urea nitrogen concentrations. The experimental design comprised a 2 × 2 factorial arrangement with different seasonality phenotypes (high and low) and two different forage-based diets, namely a medium-quality diverse treatment (Diverse) or a low-quality perennial ryegrass–white clover treatment (PRG), which were offered ad libitum. Blood and saliva samples for the determination of the PUN and SUN were evaluated monthly at civil dawn between April and September of 2022. A moderately strong relationship between the PUN and SUN was identified by simple linear regression (R^2^ = 0.65; *p* < 0.001). The interaction between diet and the date of sampling had a significant effect on the relationship between the SUN and PUN (*p* < 0.001). This interaction is likely explained by the seasonal variation in the crude protein (CP) content in the diet. The use of a mixed-model procedure improved the model fit, increasing the R^2^ by 0.12 and reducing the root mean square error by 0.341. There was no effect of seasonality on the prediction estimates of the PUN from the SUN, although a tendency for a sex–seasonality interaction was observed (*p* = 0.09). Therefore, an evaluation of the diet and date of sampling can be used to improve the reliability of the prediction estimates of the PUN from the SUN in red deer calves. Further investigation into the effects of significant factors on this relationship is required to improve the reliability of the model before the SUN can be used to predict the PUN.

**Abstract:**

Red deer (*Cervus elaphus*), like other ruminants, excrete approximately 70% of the nitrogen they ingest. Developing ways in which to reduce the rate of loss, such as manipulating the diet or selecting for efficiency of growth, requires close monitoring of the plasma urea N (PUN) concentration which, in turn, requires a simple, safe, and reliable method for collecting samples. Saliva is easier to collect than blood, but the relationship between the salivary urea N (SUN) and the PUN is not known for red deer. This was therefore evaluated in two strains of mixed-sex red deer calves (*Cervus elaphus*): a phenotype with a high seasonality of growth (H, *n* = 10) and a phenotype with a low seasonality of growth (L, *n* = 13). Both phenotypes were divided into two groups, which were each offered one of two forage-based diets ad libitum: a medium-quality diverse treatment and a low-quality perennial ryegrass–white clover treatment. Blood and saliva samples for the determination of the PUN and SUN were collected at dawn every four weeks for five months (April to September 2022). There was a strong linear relationship between the PUN and SUN in the pooled sample (R^2^ = 0.65, *p* < 0.001). The estimations of the PUN were significantly improved by adding diet and the date of sampling into the model (*p* < 0.001), but not phenotype (*p* > 0.75). SUN represents a reliable index of the PUN, and collecting saliva therefore represents a simple and inexpensive alternative to collecting blood samples in studies of nitrogen metabolism in red deer.

## 1. Introduction

The New Zealand agricultural industry is facing increasing pressure to reduce N pollution in waterways [1,2,3]. There is a superlative inefficiency of N utilisation by grazing ruminants in temperate systems [4]. At least 70% of the ingested N is not utilised to support animal production (e.g., milk and live weight gain) and is excreted mainly (over 60%) as urinary nitrogen (UN) [5]. It has been reported that in pasture-based production systems, approximately 82% of UN is discharged onto pastures as highly concentrated patches (700–1200 kg N/ha). Urinary N rapidly breaks down NH_3_, NO_3_^−^ (nitrate), and N_2_O. From this, around 20–30% is leached, and 2% is transformed into N_2_O [6].

The deer industry’s N pollution contribution is not entirely clear due to the limited data available. Such a limitation relates to the nature of deer production systems and the very nature of the animal in question. Deer, as not yet domesticated animals, are not easily accessible and can be difficult to handle in order to collect samples [7,8]. In farmed red deer (*Cervus elaphus*), venipuncture is used to measure blood metabolites, such as plasma urea N (PUN) [9]. Urea is known to equilibrate across fluids due to osmotic equilibrium, enabling relationships between UN urea and other fluids, such as milk urea N, saliva urea N, or blood urea N, to be established [10,11,12]. PUN concentrations have been shown to provide an accurate indication of UN urea [13,14].

The relationship between UN urea and other bodily fluids is typically linear and largely reflects the protein content in the diet [11]. Saliva urea N is used in human medicine as a non-invasive test to measure plasma urea N for nephropathic patients, as well as patients with chronic kidney injury, kidney disease in children, and renal failure [15,16,17,18,19,20]. This relationship has not been validated in cervids [15,16]. The main objective of our research is to evaluate the relationship between the SUN and PUN concentrations in red deer calves under differing conditions as the first step in creating an easy tool to quickly evaluate a large number of deer for UN concentration estimates. It was hypothesised that the saliva urea N (SUN) and plasma urea N (PUN) concentrations of red deer calves are, as in other animals, linearly related.

## 2. Materials and Methods

### 2.1. Experimental Design and Treatment

A total of 23 weaned red deer calves of mixed gender, including 15 yearling hinds and 8 spikers, were grouped into a 2 × 2 factorial arrangement of diet (PRG × WC and Diverse) based on nutritional treatment level and seasonality (high and low), which refers to the amplitude of the annual cycle of growth during the first year of life. The experimental unit in this trial is the animal at each point in time because the use of blocking or grouping is not practical given the area available. Animal welfare risks and undue stress caused by break-feeding deer in small areas also reduce the practicality of blocking/grouping. The experiment was conducted at Lincoln University Research Farm, Deer Unit (43°38′57″ S, 172°27′01″ E), between April and September 2022. Methods described in this paper were approved by the Lincoln University Animal Ethics Committee prior to the initiation of the experiment (AEC 2022-07).

Each group (*n* = 11 and *n* = 12) was randomly assigned a dietary treatment, specifically either a medium-quality diverse treatment (Diverse) comprising perennial ryegrass (*Lolium perenne* L.), italian ryegrass (*Lolium multiflorum*), tall fescue (*Festuca arundinacea*), meadow fescue (*Festuca pratensis*), red clover (*Trifolium pratense*), white clover (*Trifolim repens*), lucerne (*Medicago sativa*), chicory (*Cichorium intybus*), plantain (*Plantago lanceolata*), and radish (*Raphanus sativus*) or a low-quality ryegrass–white clover treatment (PRG × WC) comprising perennial ryegrass (*Lolium perenne* L.), italian ryegrass (*Lolium multiflorum*), and white clover (*Trifolim repens*). During periods of pasture deficit, from July to September, both pasture types were supplemented with baleage (ad libitum) corresponding to the diet, where the diverse diet received lucerne (*Medicago sativa*) baleage and the PRG × WC diet received perennial ryegrass-based baleage.

The PRG pasture comprised one-third of each paddock, and the diverse pastures comprised the remaining two-thirds of each paddock, with the placement of ryegrass and diverse areas alternating across each paddock to account for variation in soil types and fertility affecting plant performance. Each third of the paddock (~0.5 ha) was allocated either fortnightly or monthly behind a temporary electric break fence consisting of five reels mounted to a real stand to maximise the break size and prevent any undue stress associated with using temporary fencing equipment.

### 2.2. Care and Use of Animals

Following weaning and blocking, calves were weighed, and saliva and blood were sampled at dawn (civil twilight) for baseline levels of PUN and SUN. They were then placed on their dietary treatments and given 30 days for dietary acclimatisation. Liveweight was measured each fortnight, while saliva (saliva urea N) and blood (plasma urea N) measurements were collected from each animal every four weeks. Animal measurements were undertaken at civil twilight to reduce the effect of variation in ruminal fill on liveweight and reduce the need to fast the animals prior to weighing. Calves were mustered from their paddocks and held in two groups based on their diet groups and left undisturbed in pens in dark rooms for ~30 min prior to sampling. The Diverse diet animals were sampled first, to minimise variation, in groups of 3–4. The animals were then held in the yards, while the PRG animals were sampled using the same method. Following sampling, animals were returned to their next forage allocation.

Saliva sampling was conducted using salivette tubes (SARSTEDT, Nümbrecht, Germany). Briefly, the 38 × 10 mm cotton dental swab was taken from the salivette tube and held in the saliva pocket in the side of the mouth of the restrained deer using a long pair of clamping forceps until the swab was saturated, collecting ~1–5 mL. Blood samples were collected using 20-gauge needles and placed into 10 mL Li heparin-coated vacuettes. Following sample collection, blood and saliva were held on ice and transferred to the laboratory. Blood samples were then centrifuged at 3000× *g* for 15 min at 4 °C, and saliva samples were centrifuged at 4000× *g* for 15 min at 4 °C, as there could be a limited volume of saliva within the dental swab. Spinning the salivette tubes at a greater rate ensured that all the fluid was removed from the dental swab and that we had the best chance at collecting enough fluid for analysis. The resulting plasma and saliva were collected into 2 mL Eppendorf tubes and stored at −20 °C until analysed. Both plasma and saliva urea nitrogen were evaluated by Randox RX Daytona analyses (Randox Laboratories Ltd., Crumlin, UK).

### 2.3. Herbage

Pasture herbage mass was evaluated by cutting all plant material within a 0.2 m^2^ quadrat to ground level and drying in a 60 °C oven until achieving a constant weight. Within each treatment, 10 quadrats were collected both pre- and post-grazing to estimate pasture disappearance for each group. Plant chemical composition and botanical components were evaluated by collecting ~10–20 random hand-grab samples of herbages cut to grazing height (~3 cm) pre-grazing. Baleage was also evaluated by collecting random hand-grab samples of each bale as it was allocated to each dietary treatment.

Pasture and baleage hand-grab samples were mixed and sub-sampled for dry matter (DM%) and botanical and chemical compositions. Pasture and baleage hand-grab samples were bulked and separated into three sections to determine DM% (oven-dried at 60 °C until achieving a constant weight), chemical composition, and botanical composition. Botanical components were sorted and oven-dried to calculate relative abundance in the sward. The third sample was frozen and stored until freeze-dried, ground through a 1 mm centrifugal mill (ZM200 Retsch GmbH, Haan, Germany), and analysed for chemical components (acid detergent fibre: ADF; neutral detergent fibre: NDF; organic matter: OM; crude protein: CP; and water-soluble carbohydrates: WSCs) using near-infrared spectrophotometry (NIRS; model: FOSS NIRS Systems 5000, Minneapolis, MN, USA). Calibration equations for predicting the WSC, CP, ADF, NDF, and OM of pasture samples were developed previously by the Agriculture and Life Sciences Laboratory at Lincoln University (Christchurch, New Zealand). The R^2^ values for CP, OM, WSC, NDF, and ADF were all above 0.9, and all samples were within the calibration range (Table 1).

### 2.4. Statistical Analysis 

Measurement variables were grouped into one large dataset independent of the sampling time, dietary treatment, and animal phenotype, and a Pearsons correlation analysis was used to evaluate the relationship between PUN and SUN. PUN and SUN were then analysed using a generalised linear mixed-effects model with diet, phenotype, gender, and date of sampling as fixed effects and the individual animal as the random effect using the ‘lme’ function of the lme4 package [21] of R (r Core Team, 2018, v. 3.4.4.). Interactions between gender, date of sampling, and diet were evaluated and included in the final model (*p* < 0.05), and an ANOVA of the model was carried out using the ‘car’ package. Least square means were generated using the ‘emmeans’ package (length, R.V., using Lsmeans, CRAN, 2018 [21] of R (r Core Team, 2018, v. 3.4.4.). Upon the significance of the ANOVA, the means were separated using pairwise contrasts. Differences were declared significant if *p* < 0.05 and tendencies were 0.05 < *p* < 0.1.

## 3. Results

### 3.1. Diet Composition and Nutritional Value

The dry matter content of herbage and baleage in the PRG was 34% greater than that of the diverse equivalents, as seen in Table 2. The Diverse herbage had a greater feeding value than the PRG, with the PRG containing a 6% higher content of weeds and a 16% higher content of dead material. Table 3 shows that crude protein was highly variable between treatments and seasons. Digestibility and organic matter were similar between treatments, with organic matter remaining similar across the five-month period, while digestibility appeared to be highly variable between seasons. The metabolisable energy content (MJ ME/kg DM) varied between seasons, although the Diverse forage had a greater metabolisable energy content than the PRG treatment at each corresponding period. The digestibility of the PRG baleage was also variable across seasons, with greater digestibility than the Diverse baleage between June and August, while the Diverse baleage had greater digestibility in the August–September period. The greatest difference in digestibility was seen in the June–July period. The protein content of diverse baleage was greater than that of the PRG baleage in the June–July period and the August–September period, with similar magnitudes of difference. However, in the July–August period, there was no significant difference between the protein content of the PRG and Diverse baleage.

### 3.2. Relationship between Plasma Urea N and Saliva Urea N

**Figure 1:**
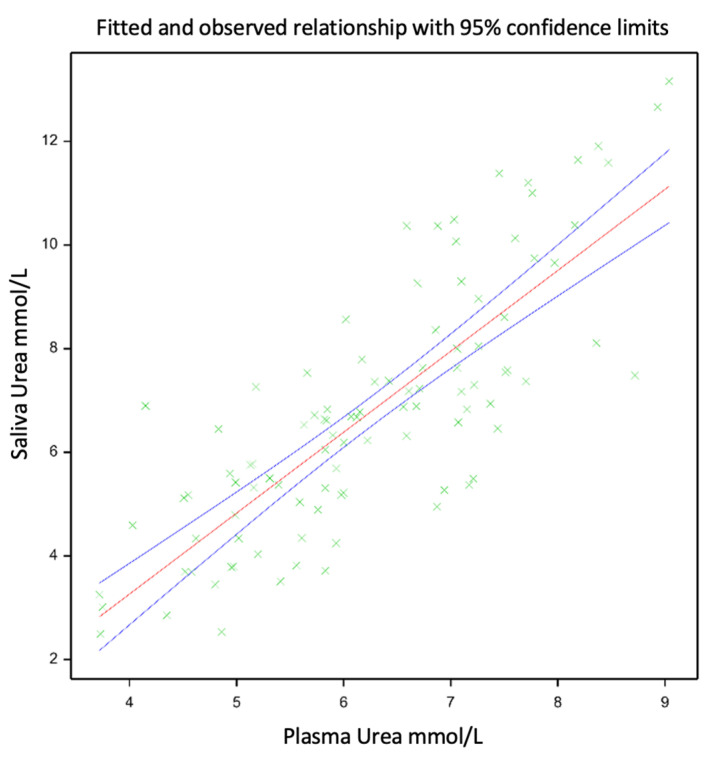
Relationship between SUN and PUN for growing red deer calves selected for different phenotypes (divergent amplitude seasonal growth) under two nutritional level treatments, either ‘Diverse’ or ‘PRG’, respectively, given by either low-quality ryegrass–white clover swards and ryegrass-based baleage or medium-quality diverse swards and lucerne baleage. The green plus signs within this figure indicate datapoints, while the red line indicates the relationship between the datapoints, and the blue lines show the confidence interval for this relationship.

The means of the PUN and SUN were similar, 6.26 and 6.79 mm/L, respectively, showing a linear relationship (SUN = −2.978 + PUN × 1.561; Figure 1), with a Pearson correlation coefficient of 0.8 and an R^2^ of 0.65 (*p* < 0.001). The linear relationship between the PUN and SUN is presented in Figure 1. An evaluation of the relationship between the PUN and SUN was carried out using the mixed model described previously, which improved the model fit, increased the adjusted R^2^ from 0.65 to 0.77, and reduced the root mean square error from 0.734 to 0.393.

**Figure 2:**
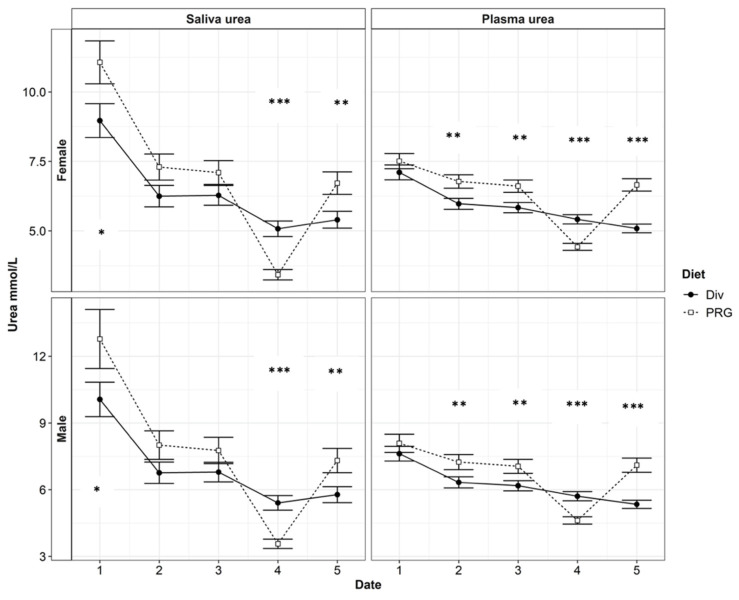
The plasma urea nitrogen (PUN) and saliva urea nitrogen (SUN) contents in red weaner deer each month between April and September. Date refers to each monthly sampling between April and September (labelled 1:5, respectively). Significance of Diet (either a diverse pasture, Div, or a ryegrass-based diet, PRG) is indicated by *p* < 0.05: *, *p* < 0.01: **, or *p* < 0.001: ***.

The Diverse diet reduced the PUN compared with that of the PRG treatment, while the dietary means of the SUN were similar between diets. A significant interaction between diet and month was detected for both the PUN and SUN (*p* < 0.001; Table 4). In August, the PRG diet reduced both the PUN and SUN compared with the Diverse treatment. In September, both the PUN and SUN increased in the PRG diet and were greater than that observed in calves given the Diverse diet. The SE for the SUN was twice the standard error of the PUN (Table 4), as depicted in Figure 2, which presents greater variability in the SUN values compared to the PUN values. The effect of gender or seasonality (calf phenotype) on either the PUN or SUN was not significant (*p* > 0.05, Table 4), but a tendency for a gender–seasonality interaction was identified (*p* = 0.09).

The high-seasonality yearling hinds had lower PUN (6.29 and 5.64 ± 0.221 mmol/L for spikers and yearling hinds, respectively) and SUN (7.0 and 6.0 ± 0.353 mmol/L for spikers and yearling hinds, respectively) concentrations compared with the high-seasonality spikers, while the PUN (6.42 and 6.39 ± 0.171) and SUN (6.35 and 6.35 ± 0.234) concentrations of the low-seasonality yearling hinds and spikers were similar (*p* > 0.1). Month and diet had a significant influence on the relationship between SUN and PUN. Including the interaction effects of month and diet can improve the R^2^ to ~0.75 (*p* < 0.001). The urea N levels in all categories of the low-quality ryegrass–white clover treatment were significantly lower in August (Month 4). The PUN levels of deer on the Diverse diet did not decline significantly in August. For all treatments, there was an overall decrease in the urea N levels throughout the trial, with the lowest urea N levels being found in August and September (month 4 and month 5). The N contents of the SUN and PUN over time, as grouped by gender, are presented in Figure 2.

## 4. Discussion

### 4.1. The Relationship between Saliva Urea N and Plasma Urea N

The objective of our research was to evaluate the relationship between SUN and PUN concentrations in red deer fawns with high- and low-seasonality growth pattern phenotypes that were fed either a moderate-quality diverse sward or a low-quality ryegrass–white clover sward. The results of this study support our hypothesis as they indicate that there was an acceptable positive linear relationship between SUN and PUN concentrations (*p* < 0.001; R^2^ = 0.65). Estimations of the PUN using the SUN can be improved, though, by adding other effects to the model, such as diet, date and seasonality. Our results show that the relationship between the PUN and SUN was moderately strong (R^2^ = 0.65) when using a simple linear model. Using this model, the SUN can be estimated using the PUN, and the PUN can also be estimated using the SUN. The reliability of these estimations is limited by the strength of the relationship. As there is only a moderately strong linear relationship between plasma and saliva urea N, this relationship equation cannot reliably predict PUN levels based on the saliva urea N levels. The improved model fit provided by the mixed-model procedure increased the adjusted R^2^ value by 0.12 and reduced the root mean square error by 0.341 due to the inclusion of fixed effects, such as diet, date and seasonality. Therefore, an evaluation of the diet and date of sampling can be used to improve the reliability of the model to predict the PUN from the SUN concentration. 

### 4.2. Significant Effects on the Relationship between Saliva Urea N and Plasma Urea N

The interaction between diet and sampling date had a significant effect on the relationship between the SUN and PUN concentrations. Including the interaction effects of month and diet increased the R^2^ value to ~0.75 (*p* < 0.001). This interaction is likely explained by the crude protein (CP) content in the diet. The CP content of the Diverse treatment was similar in April, June, and July. The CP content of the PRG diet was lower in August than the CP content of the Diverse diet. However, in September, the CP content of the diverse diet was less than that of the PRG treatment. These results are reflected in the SUN and PUN levels presented in Figure 2. While there was no dietary difference between the SUN and PUN levels in June and July, the dietary differences observed in the PUN and SUN levels in August and September appear to reflect the changes in the CP contents in the diets during these months. In August, the animals undergoing the PRG diet treatment had lower SUN and PUN levels compared with the animals undergoing the Diverse diet treatment. In September, when the Diverse treatment had lower CP levels, the animals also had lower SUN and PUN levels than those fed the PRG diet. This is consistent with the findings of [22], which revealed that the CP level within the diet is strongly correlated to the urinary N output of dairy cows. The crude protein levels in pastures change throughout the year; typically, they increase over the warmer summer months and decline through the colder winter months [23]. Therefore, an evaluation of the CP contents in the animals’ diets throughout the year can increase the reliability of PUN predictions when using the SUN.

The dietary differences observed in the PUN and SUN levels between each month may also be partially explained by the presence of secondary metabolites within the forage. Different types of plant secondary metabolites, such as phenolics, terpenes, glucosinolates, and alkaloids, all affect the animals differently, eliciting differing responses. The Diverse forage has a large variety of plants, including grasses, legumes, herbs, forbs, and brassicas. This variety of plants will therefore likely contain a range of plant secondary metabolites. Seasonal changes, both within the individual plant species and in the forage, as the availability of plants within the forage changes, will likely cause changes in the available plant secondary metabolites, causing a response within the animal. Polyphenols, found within chicory, have been reported to show seasonal variation and influence the production of proline-based salivary proteins [24].

There was a significant effect of a gender and seasonality interaction on the SUN and PUN levels. However, the effect of this interaction is outside the scope of our study. Figure 2 presents both the SUN and PUN levels in spikers, suggesting that, although the PUN and SUN levels were numerically greater than that of the yearling hinds, they were statistically similar (*p* > 0.1). The tendency of there being an interaction between gender and seasonality may support the natural effect of sexual dimorphism, and in this case, it is more marked between the high-seasonality spikers and yearling hinds. The effects of sexual dimorphism on red deer may allow for greater N use efficiency of the males in comparison to females [25]. Spikers have a greater plane of nutrition and metabolic requirement than yearling hinds. This means that they will typically use a comparatively greater proportion of N from the forage on maintenance and growth [26]. Animals with a greater N demand tend to utilise N much more efficiently. More efficient N utilisation allows for faster animal growth from a similar or lower N input, and therefore, a potential reduction in the negative environmental N output is achieved [27], as N is diverted to tissue rather than excretion through urine or faeces. However, N intake was not measured in this study; therefore, these speculations should be taken with caution. The interaction between gender and seasonality observed for the PUN and SUN concentrations requires further investigation.

### 4.3. Estimation of Plasma Urea Nitrogen from Saliva Urea Nitrogen

The main objective of this study was to determine whether an easy-to-measure animal variable like the SUN could be used as a tool to quickly evaluate a large number of deer for UN concentration estimates. Arguably, the handling of deer, especially mature animals, is not easy. Blood sampling, unless conducted by experienced personnel, often requires 3–4 people and can create a stressful environment for the animal. Therefore, SUN sampling might emerge as a more practical alternative. Figure 2 shows that there was a varying magnitude of difference between the SUN and PUN levels between each sample period, with the greatest difference occurring in April. To increase the reliability of PUN estimates using the SUN, significant factors, such as the interaction between diet and sampling date and the gender–seasonality interaction, need to be considered and adapted for the predictive model.

The technique and methodology for collecting saliva samples for determining the SUN in deer have not been well described in the literature and still need further considerations and methodological refining. The exact anatomical location of the oral cavity, the time required for sufficient swab saturation, and the time of day are all factors that may impair the collection of a representative sample. The anatomy of the salivary glands in red deer differs from that of other domesticated ruminants as deer have three saliva glands made up of one or more connected saliva ducts, and each saliva gland may excrete saliva at different rates with differing UN concentrations [28]. Secretions from the parotid gland contains a significantly greater urea concentration than secretions from both the submandibular gland and the sublingual gland [16]. The parotid gland is the heaviest saliva gland in relation to the animal’s body weight, and it is vital in ruminal digestion regulation [29]. The variation in UN between saliva glands may explain some of the variation in the SUN concentrations in the present study; therefore, a further evaluation of the methodology used to collect saliva samples is needed.

To improve the accuracy of predicting the PUN by SUN, i.e., reducing the SUN variability, a more specific sampling site should be used, such as the saliva pocket within the right side of the jaw. This will most likely reduce the between-animal variation as well. The sampling method used in this trial could also be adapted more closely to the methods of trials looking at tannin-binding properties, such as the one by Fickel [30], which uses collection catheters to collect saliva from the parotid gland. Samples of mixed saliva were also collected and compared to parotid gland saliva for reliability in relation to tannin-binding properties. Fickel [30] also reported that using saliva exclusive to the parotid gland presented an increased number of tannin-binding properties. However, the use of collection catheters is more invasive than regular saliva sampling or blood sampling, as animals need to be euthanised before the catheter can be inserted. Therefore, this method is not practical for commercial-scale SUN determination. Saliva samples are also often contaminated by ingesta and digesta and need to be carefully examined before an SUN analysis [31]. A potential alternative to reduce the contamination of digesta is to take the sample before their dawn grazing bout, as ruminants generally fast overnight. Contamination of saliva may also be reduced if sampling occurs during idling times, as residues of the rumination boli may be potentially less [32]. However, individual animals often ruminate and idle during different periods throughout the day. Overall, sampling using mixed saliva is less stressful and safer, especially when handling larger stags, spikers, or hinds [9]. To reliably replace plasma with saliva sampling, further development to this model is required using a larger sample size and investigating significant effects. With a larger number of animals, this sampling technique still has the potential to become a reasonably non-invasive and easy tool to quickly evaluate a large number of deer for UN concentration estimates [15,16,17,18,19,20]. 

## 5. Conclusions

The implications of these findings are promising for the New Zealand deer industry. There is a significant linear relationship between the SUN and PUN levels in red deer calves. Therefore, the SUN can be used to estimate the PUN, and thereby, the potential difference in individual animal UN excretion can be calculated through saliva sampling. Using a simple linear regression, the SUN explained 65% of the variation in the PUN; therefore, to increase the reliability of PUN estimates, other factors/effects need to be considered in the predictive model, e.g., diet by sampling date, as demonstrated in the present study. When the interaction between month and diet was included in the model, ~75% of the variation in the PUN was explained by the SUN. Deer are not easy to handle; therefore, with careful sampling methods, determining the SUN may emerge as an easy and practical sampling technique to evaluate large numbers of mature red deer.

## Figures and Tables

**Table 1:** Lincoln University Agriculture and Life Sciences Laboratory NIRS Calibration Equations for Dry Green Matter.

Constituent	N	Mean	SD	Established Minimum	Established Maximum	SEC	RSQ	SECV	1-VR
Protein	250.00	16.09	7.01	0.00	37.11	0.86	0.99	1.04	0.98
DOMD	247.00	68.47	13.59	27.69	109.25	2.36	0.97	2.83	0.96
OM	250.00	89.40	3.54	78.79	100.02	1.19	0.89	1.39	0.85
DMD	250.00	72.81	13.23	33.11	112.51	2.15	0.97	2.44	0.97

**Table 2:** Botanical composition of herbage of each dietary treatment (Diverse and PRG) offered to red deer calves of different phenotypes for growth pattern.

Treatment	Grasses	Legumes	Forbs	Brassicas	Weeds	Dead Material
Diverse	25.9%	20.7%	8.2%	13.4%	2.3%	29.5%
PRG	24.3%	22.1%	0.0%	0.0%	17.8%	35.7%

**Table 3:** Chemical composition and nutritive value of herbage and baleage offered to growing red deer calves selected for different phenotypes (divergent amplitude seasonal growth) under two nutritional level treatments, either ‘Diverse’ or ‘PRG’, respectively, given by either low-quality ryegrass–white clover swards and baleage or medium-quality diverse sward and lucerne baleage.

Variable	May–June				June–July								July–August								August–September			
Nutritive Value	Diverse	SE	PRG	SE	Diverse	SE	Diverse Baleage	SE	PRG	SE	PRG Baleage	SE	Diverse	SE	Diverse Baleage	SE	PRG	SE	PRG Baleage	SE	Diverse Baleage	SE	PRG Baleage	SE
Protein g/kg DM	13.41	0.30	14.06	0.54	16.26	0.25	15.28	1.37	13.50	<0.01	8.49	0.82	21.18	0.03	15.69		22.13	0.50	15.21	0.61	16.62	0.37	8.39	0.41
Digestibility (DOMD) g/kg DM	60.80	0.48	56.08	0.83	65.23	0.53	45.52	3.05	61.30	0.24	57.74	4.33	71.96	0.77	56.82		69.54	0.54	66.41	1.42	61.16	6.51	51.14	1.26
MJ ME/kg DM	9.63	0.12	9.17	0.09	10.12	0.02			9.71	0.01	66.41		11.02	0.02			10.79	0.22						
OM g/kg DM	93.04	0.56	93.46	0.18	91.67	0.13	92.41	0.02	93.01	0.18	94.42	0.07	90.69	0.06	91.83		90.92	0.53	90.46	0.39	90.73	0.44	93.59	0.35
DM g/kg DM	25.00	6.79	29.10	8.03	24.30	7.13	29.20	0.44	40	6.65	66.40	0.71	20.30	68.31	41.40	0.03	17.80	3.55	56.90	0.44	47.80	1.04	65.9	0.37
Average % DM Consumed	43%		45%		53%				15%				73%											

**Table 4:** Mean differences (±standard error) between saliva urea nitrogen (SUN) and plasma urea nitrogen (PUN) of high- or low-seasonality red weaner deer fed either perennial ryegrass-based diet (PRG) or diverse pasture species diet (Diverse). Significance of interaction terms between diet and month (D × M) and gender and seasonality (G × S) are also presented.

*p*-Value *								
	PRG	Diverse	Diet	Month	Gender	Seasonality	D × M	G × S
SUN (mmol/L)	6.42 ± 0.231	6.39 ± 0.187	0.02	***	0.23	0.75	***	0.09
PUN (mmol/L)	6.37 ± 0.155	5.97 ± 0.114	**	***	0.21	**	***	0.09

*p* < 0.05 = *; *p* < 0.01 = **; *p* < 0.001 = ***.

## Data Availability

The raw data supporting the conclusions of this article will be made available by the authors on request.
